# Retained Activity of an O25b-Specific Monoclonal Antibody against an Mcr-1-Producing Escherichia coli Sequence Type 131 Strain

**DOI:** 10.1128/AAC.00046-18

**Published:** 2018-06-26

**Authors:** Luis M. Guachalla, Katharina Ramoni, Cecilia Varga, Michele Mutti, Akela Ghazawi, Tibor Pál, Eszter Nagy, Ágnes Sonnevend, Gábor Nagy, Valéria Szijártó

**Affiliations:** a Arsanis Biosciences, Vienna, Austria; b College of Medicine and Health Sciences, United Arab Emirates University, Al Ain, United Arab Emirates

**Keywords:** Escherichia coli ST131, *mcr-1*, monoclonal antibodies

## Abstract

Plasmid-encoded colistin resistance is emerging among extraintestinal pathogenic Escherichia coli strains, including those of the epidemic clone sequence type 131 (ST131)-H30. Mcr-1 transfers a phosphoethanolamine to the lipid A portion of lipopolysaccharide (LPS), conferring resistance to polymyxins. We investigated whether this modification changed the activity of the monoclonal antibody ASN-4, specific to the O25b side chain of ST131 LPS. We confirmed that, unlike colistin, ASN-4 retained its bactericidal and endotoxin-neutralizing activities and therefore offers a treatment option against extremely drug-resistant ST131 isolates.

## TEXT

Colistin (polymyxin E) and polymyxin B are considered to be last-resort antibiotics against multidrug-resistant (MDR) Gram-negative bacteria. With the spread of carbapenem-resistant pathogens, the use of colistin has increased, leading to the emergence of colistin resistance (1). Recent reports on the plasmid-mediated colistin resistance determinants, *mcr-1* and allelic variants thereof (2–4), underline the importance of transmissible resistance genes in the rapid emergence of colistin resistance (1). The acquisition of *mcr-1* by isolates belonging to globally spread MDR high-risk clonal lineages, including Escherichia coli clone sequence type 131 (ST131)-H30, poses a major threat to the health care system.

The gene product of the *mcr* alleles transfers a phosphoethanolamine residue to the lipid A moiety of the lipopolysaccharide (LPS) (4), i.e., the molecular target of polymyxins. The modified lipid A has much lower affinity for colistin and related polymyxins, resulting in reduced activities for these drugs. Beyond conferring resistance to polymyxins, Mcr-1 was also shown to alter the susceptibility of bacteria to antimicrobial peptides (5) and unrelated antibiotics (6). Additionally, a high level of Mcr-1 expression was reported to compromise growth rate, fitness, and outer membrane structural integrity through the integration of Mcr-1 into the membrane, as well as its enzymatic activity, i.e., the modification of the lipid A (6).

We have previously reported humanized monoclonal antibodies (7) targeting the lipopolysaccharide O25b antigen associated with the ST131-H30 clone (8), the most prevalent extraintestinal pathogenic E. coli (ExPEC) lineage, responsible for a wide range of infections. We demonstrated that these monoclonal antibodies (MAbs) have complement-dependent bactericidal, opsonophagocytic, and endotoxin-neutralizing activities that contribute to protection in murine models (9).

In the current study, we show that all three mechanisms of action of an O25b-specific MAb, ASN-4, were retained against E. coli ST131-H30 isolates producing Mcr-1. ASN-4 shares the complementarity-determining region (CDR) sequences of the previously published MAb A1124 (9) and therefore has identical binding specificity and affinity (with only two amino acid changes in the framework region).

To compare the potency of ASN-4 against Mcr-1-producing versus -nonproducing strains, an isogenic ST131-H30 pair was generated. The open reading frame of *mcr-1* was cloned from the bloodstream isolate ABC149 (10) into the SalI and EcoRI sites of plasmid pBAD322C (pBAD::*mcr-1*). Strain 81009 was transformed with pBAD::*mcr-1* (VSZ197) or with the empty vector (VSZ198). The expression of *mcr-1* was induced with 0.02% arabinose, which resulted in a 4-fold elevation in the colistin MIC in VSZ197 compared to VSZ198.

**FIG 1:**
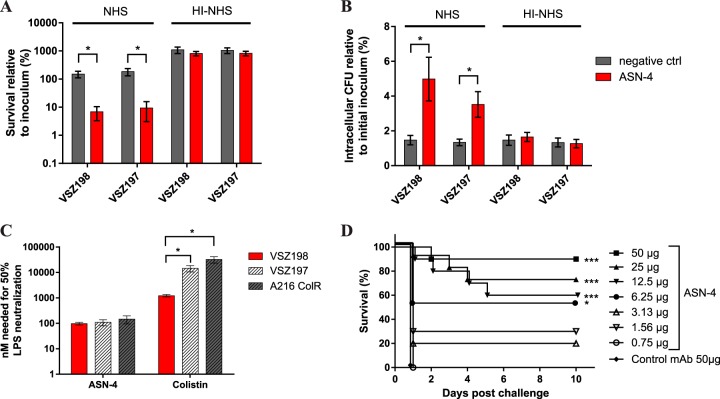
Activity of the ASN-4 MAb *in vitro* and *in vivo*. (A) Complement-mediated bactericidal activity of ASN-4 against an isogenic *mcr-1*-expressing (VSZ198) and -non-expressing (VSZ197) strain pair in 50% human serum after 3 h of incubation. The combined results of 3 independent experiments are shown. (B) Opsonophagocytotic uptake of the isogenic strain pair by RAW 264.7 cells mediated by ASN-4 in the presence of 5% human serum. The combined results of 6 independent experiments are shown. (C) *In vitro* neutralization of endotoxin activity of purified LPS from VSZ197 and VSZ198 or the colistin-resistant clinical isolate ABC149 as measured by a cell-based TLR4 reporter assay. The combined results of 5 independent experiments are shown. (D) Prophylactic efficacy of ASN-4 in a murine endotoxemia model. BALB/cJRj mice were immunized with the indicated doses of MAbs and subsequently challenged with 0.5 ng purified LPS extracted from strain ABC149. The results from two independent experiments with groups of 5 mice each (total of 10 mice per dose) are shown. Data shown in panels A to C are presented as the mean ± standard error of the mean (SEM), and groups were compared with Student *t* test, while on panel D, Mantel-Cox survival curves were analyzed with the log-rank test. *, *P* < 0.05; ***, *P* < 0.001. In all experiments, an isotype-matched (human IgG1) MAb with irrelevant specificity was used as a negative control. NHS, normal human serum; HI, heat inactivated.

The complement-dependent bactericidal and opsonophagocytic activities of ASN-4 were measured as described previously (9). In 50% human serum as a complement source, ASN-4 mediated significant killing of both strains (Fig. 1A). Similarly, in the presence of active complement, ASN-4 significantly increased the uptake of either strain by murine macrophages (compared to a control IgG) (Fig. 1B). No significant difference was observed between the activities against *mcr-1*-expressing and -nonexpressing strains in any of these assays. These data confirm that the expression of *mcr-1* did not influence the binding of ASN-4 to the LPS O antigen or the integration of the complement membrane-attack-complex (MAC) into the structurally altered outer membrane.

The proinflammatory activity of the LPS is primarily mediated by its lipid A portion (i.e., the endotoxin). Although ASN-4 does not directly target the lipid A moiety, but rather the carbohydrate part of LPS, we were able to show that A1124, the parent MAb, neutralized the proinflammatory activity of LPS both *in vitro* and *in vivo* (9). This activity can originate from steric blocking of the lipid A binding to its receptor complex or from the stabilization of the supramolecular micelle structure of LPS that may be affected by the *mcr-1*-encoded modification. Therefore, it was of particular interest to evaluate whether the neutralizing activity of ASN-4 against LPS altered by Mcr-1 would be affected. The reporter cell line HEK-Blue hTLR4 (InvivoGen) was used to measure endotoxin-induced Toll-like receptor 4 (TLR-4) signaling. Cells were incubated with purified LPS extracted from VSZ197, VSZ198, or the *mcr-1*-expressing clinical ST131-H30 isolate, ABC149, in the presence of ASN-4 or the endotoxin-neutralizing antibiotic, colistin. The signal triggered by the different LPS samples alone was adjusted to be comparable. Next, we determined the MAb or colistin concentration required for 50% reduction (50% effective concentration [EC_50_]) of the TLR-4 activation signal (Fig. 1C). While the neutralizing potencies of colistin against strains VSZ197 and ABC149 were 12- and 27-fold lower, respectively, than that against the colistin-susceptible strain VSZ198, ASN-4 had comparable LPS-neutralizing capacity against all E. coli ST131 strains independent of the presence of *mcr-1*.

To further corroborate this finding, we tested the efficacy of ASN-4 in a mouse endotoxemia model. Animal experiments were reviewed and approved by Arsanis' Animal Welfare and Ethics Committee and were performed according to Austrian law (BGBl. I no. 114/2012, https://www.ris.bka.gv.at/Dokumente/BgblAuth/BGBLA_2012_I_114/BGBLA_2012_I_114.html), as approved by the respective competent authority (Magistratsabteilung 58, Vienna, Austria). Six- to eight-week-old female BALB/cJRj mice were sensitized to endotoxin (11), passively immunized with different doses of ASN-4 or a control MAb, and 24 h later challenged with a lethal dose of purified LPS extracted from strain ABC149. Prophylactically administered ASN-4 provided significant protection at a dose of 6.25 μg/animal (∼300 μg/kg of body weight) or higher (Fig. 1D). This efficacy is comparable to that observed previously against a colistin-susceptible clinical isolate (9).

In conclusion, we demonstrate that all three mechanisms of action, i.e., complement-mediated killing, opsonophagocytosis, and endotoxin neutralization, of the O25b-specific MAb ASN-4 have been retained against an *mcr-1*-positive colistin-resistant ST131-H30 strain. Consequently, the use of such LPS O-antigen-targeting antibodies can be considered an alternative strategy to combat MDR infections, even against the emerging *mcr-1*-positive isolates.
